# Copper‐Mediated Radiofluorination of the Excitatory Amino Acid Transporter 2 (EAAT2)–Targeted PET Tracer [^18^F]RP‐115: Alternate Synthetic Method

**DOI:** 10.1002/jlcr.70044

**Published:** 2026-10-28

**Authors:** Sang Hee Lee, Joseph Blecha, Jung Min Kim, Ryan Michael Nillo, Robert R. Flavell, Chih‐Kai Chao, John R. Forsayeth, John M. Gerdes, Henry F. VanBrocklin, David M. Wilson

**Affiliations:** ^1^ Department of Radiology and Biomedical Imaging University of California, San Francisco San Francisco California USA; ^2^ Rio Pharmaceuticals, Inc. San Francisco California USA

**Keywords:** [^18^F]RP‐115, copper‐mediated radiofluorination, excitatory amino acid transporter 2, prodrug, radiotracer

## Abstract

Excitatory amino acid transporter 2 (EAAT2) is a key regulator of extracellular glutamate in the central nervous system. Dysregulated EAAT2 is implicated in neurodegenerative diseases such as Alzheimer's disease (AD) and amyotrophic lateral sclerosis (ALS), highlighting EAAT2 as a potential target for PET imaging. We previously reported a multistep radiosynthesis of [^18^F]RP‐115, a CNS‐permeable prodrug that undergoes esterase‐mediated hydrolysis in the CNS to yield the active EAAT2‐binding carboxylate. Motivated by recent advances in copper‐mediated radiofluorination (CMRF), we sought to develop a more efficient late‐stage radiosynthesis of [^18^F]RP‐115. A two‐step radiosynthesis of [^18^F]RP‐115 using CMRF provided [^18^F]RP‐115 in high radiochemical yield (52.8% ± 2.1 %, decay‐corrected from starting [^18^F]fluoride ion, *n* = 4), high molar activity (355 ± 61 GBq/μmol), and high radiochemical purity (> 99%) with a total synthesis time of 70 min. Chiral HPLC analysis demonstrated that the configuration of the aspartic acid residue remained intact without racemization during both the CMRF and trifluoroacetic acid mediated deprotection. We developed an efficient radiosynthesis of [^18^F]RP‐115 via CMRF, achieving high radiochemical yield and molar activity. This efficient synthesis may serve as a basis for the future implementation of a CMRF‐based automated radiosynthesis of [^18^F]RP‐115.

## Graphical abstract

An efficient two‐step radiosynthesis of [^18^F]RP‐115 was developed using copper‐mediated radiofluorination (CMRF) followed by deprotection. In a total 70‐min synthesis time, this approach provided [^18^F]RP‐115 in high radiochemical yield (52.8% ± 2.1%, decay corrected from stating [^18^F]fluoride ion) with high molar activity (355 ± 61 GBq/μmol) and radiochemical purity (> 99%), without racemization. This simplified method may offer an alternative synthetic route to facilitate future automated radiosynthesis of [^18^F]RP‐115.

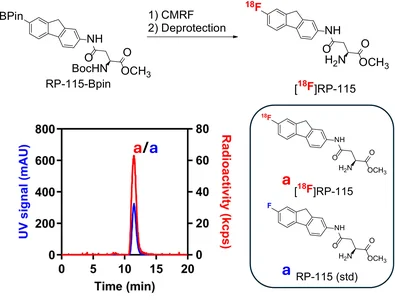

**Abbreviations**
AD: Alzheimer's disease
ALS: amyotrophic lateral sclerosis
Boc: tert‐butyloxycarbonyl
Bpin: boronic acid pinacol ester
CMRF: copper‐mediated radiofluorination
CNS: central nervous system
DCM: dichloromethane
DIPEA: diisopropylethylamine
EAAT2: excitatory amino acid transporter 2
EtOAc: ethyl acetate
GLP: good laboratory practice
HATU: hexafluorophosphate azabenzotriazole tetramethyl uronium
HD: Huntington's disease
HPLC: high‐performance liquid chromatography
MeCN: acetonitrile
PD: Parkinson's disease
SUV: standardized uptake value
TFA: trifluoroacetic acid

## 1 Introduction

Regulation of extracellular glutamate is essential for neurotransmission in the central nervous system (CNS) [1]. The excitatory amino acid transporter 2 (EAAT2, human isoform) plays an important role in removing extracellular glutamate from the synaptic cleft in the CNS [2]. Downregulated EAAT2 expression or activity leads to abnormal extracellular glutamate accumulation and subsequent glutamatergic excitotoxic neuronal damage [3, 4]. Thus, EAAT2 is implicated in the pathology of neurodegenerative diseases such as Alzheimer's disease (AD), Parkinson's disease (PD), Huntington's disease (HD), and amyotrophic lateral sclerosis (ALS) [5, 6, 7].

We recently reported a novel fluorine‐18 labeled aspartic acid analog, methyl‐N‐4‐(7‐fluoro‐9H‐fluoren‐2‐yl)asparaginate ([^18^F]RP‐115), for PET imaging of EAAT2 density [8, 9]. To improve the limited CNS penetration of the polar and charged aspartic acid moiety, [^18^F]RP‐115 was designed as a CNS‐permeable prodrug by masking the carboxylic acid as a methyl ester [10]. This brain‐targeted drug delivery strategy facilitates blood–brain barrier penetration of the EAAT2‐binding radiotracer, which subsequently undergoes esterase‐mediated hydrolysis to give the active carboxylic acid form in the CNS [11]. We previously demonstrated robust CNS uptake (SUV > 1) and EAAT2 target specificity after intravenous injection of [^18^F]RP‐115 in rodents and nonhuman primates [8, 9, 10]. Based on the safety profile confirmed by a GLP‐compliant toxicology study, using a nonradioactive RP‐115 standard, a first‐in‐human dosimetry study of [^18^F]RP‐115 was successfully conducted in healthy volunteers [12].

The initial radiosynthesis of [^18^F]RP‐115 was achieved via electrophilic radiofluorination of a tributylstannyl precursor using carrier‐added [^18^F]F_2_ gas, resulting in low radiochemical yield (d.c., ~3.5%) and low molar activity (89 MBq/μmol) product within a total synthesis time of approximately 150 min [10] (Figure 1A). More recently, a multistep alternative radiosynthetic route for [^18^F]RP115 was developed via nucleophilic radiofluorination with no‐carrier‐added [^18^F]fluoride ion, providing substantially higher molar activity and improved radiochemical yield suitable for clinical translation [12]. Starting from commercially available 2,7‐dinitro‐9‐fluorenone (5 g, < $100), the nucleophilic radiofluorination of [^18^F]RP‐115 comprised multiple steps: (1) radiofluorination, (2) reduction, (3) coupling with a protected aspartic acid derivative, and (4) Boc deprotection. Under the fully optimized automated protocol, both radiochemical yield (13.7% ± 7.1%) and molar activity (112 ± 81 GBq/μmol) were improved with a comparable total synthesis time (163 ± 15 min) (Figure 1B).

**FIGURE 1:**
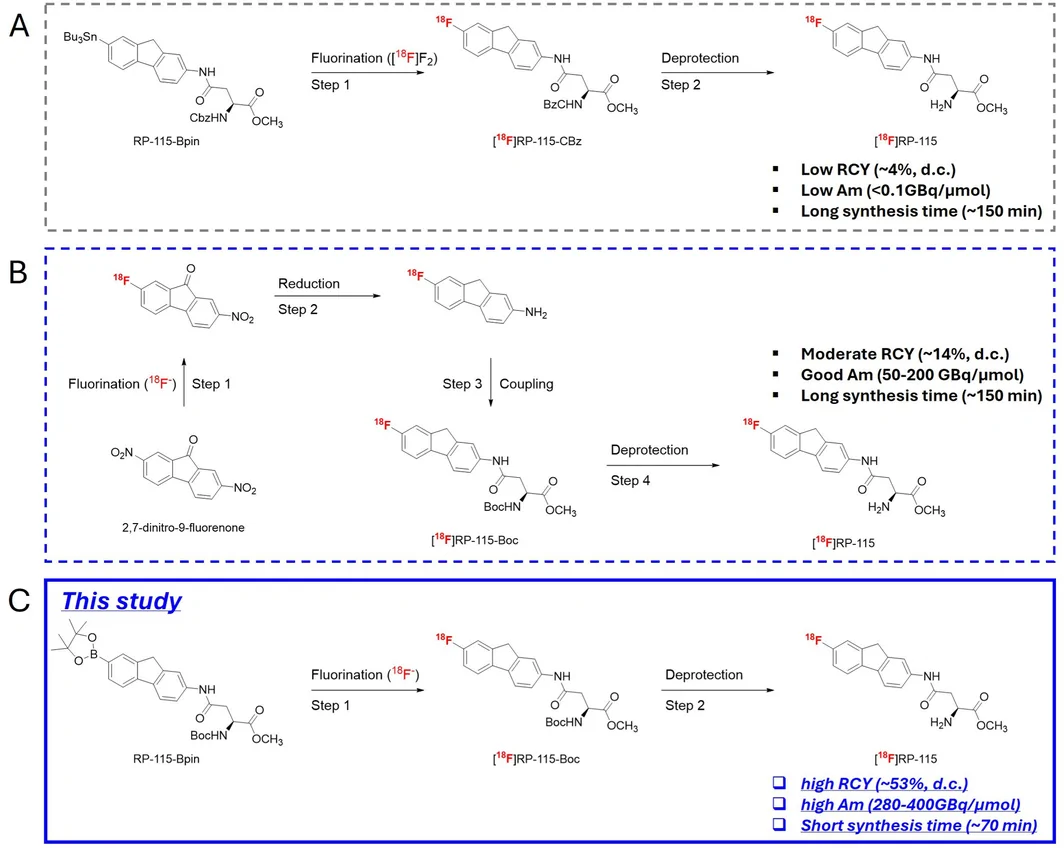
Radiosynthetic approaches for [^18^F]RP‐115. (A) Initial approach of [^18^F]RP‐115 radiosynthesis via carrier‐added electrophilic radiofluorination, resulting in a low radiochemical yield (RCY) and molar activity (A_m_). (B) The fully automated radiosynthesis of [^18^F]RP‐115 via no‐carrier‐added nucleophilic radiofluorination, resulting in an improved RCY and A_m_. (C) Herein, we report the CMRF‐based simplified method as a potential alternative route for [^18^F]RP‐115 radiosynthesis. The manual radiosynthesis, consisting of CMRF followed by Boc deprotection, provided a high radiochemical yield and high molar activity within a short synthesis time.

Meanwhile, the field of radiofluorination chemistry has evolved, particularly with respect to aromatic ^18^F‐fluorination [13, 14, 15]. The development of radiosynthetic methods for fluorine‐18 labeled dihydroxyphenyl alanine ([^18^F]FDOPA), which is one example of a challenging electron‐rich arene, exemplifies the historical progression of aromatic radiofluorination chemistry [16]. [^18^F]FDOPA is a radiopharmaceutical for imaging dopaminergic pathways in several brain diseases, including PD [17]. Initial production methods in the early 1980s employed electrophilic aromatic radiofluorination using carrier‐added [^18^F]F_2_ gas, enabling production of [^18^F]FDOPA in millicurie quantities suitable for PET imaging [18]. However, this approach was limited by low radiochemical yield, low molar activity, and poor regioselectivity resulting in formation of different fluorinated regioisomers [18, 19]. Radiofluorodestannylation using a trimethylstannyl precursor enhanced regioselectivity; however, the low molar activity (~10 MBq/μmol) remained a concern [20, 21]. To improve its radiosynthesis, considerable efforts were directed toward improving electrophilic [^18^F]F_2_ production [22] and designing alternative multistep radiosynthetic routes using the [^18^F]fluoride ion [23]. Newer advanced late‐stage aromatic radiofluorination methods using hypervalent iodine precursors such as iodonium ylides [24] and iodonium salts [25] were developed. More recently, copper‐mediated radiofluorination (CMRF) has emerged as a robust aromatic radiofluorination technique for the efficient radiosynthesis of brain‐targeted radiotracers such as [^18^F]FDOPA [26], [^18^F]flumazenil [27], and [^18^F]BS224 [28]. A fully automated high molar activity radiosynthesis of [^18^F]FDOPA via CMRF has been well established [29]. Additionally, Scott, Sanford, and co‐workers reported the efficient synthesis of diverse ^18^F‐labeled analogs, including amino acids and peptides, via sequential Miyaura borylation followed by CMRF from stable and readily available (hetero)aryl halides, further highlighting the robustness of CMRF as a versatile aromatic radiofluorination method [30].

Motivated by recent advances in CMRF‐based aromatic radiofluorination, we aimed to establish an alternative radiosynthetic route for late‐stage fluorination of [^18^F]RP‐115 (Figure 1C). We designed a boronic acid pinacol ester (Bpin) precursor to develop late‐stage CMRF of [^18^F]RP‐115. The radiosynthesis of [^18^F]RP‐115 was established in two steps, consisting of (1) CMRF and (2) Boc deprotection. Each step was investigated systematically, and the overall sequence was finally integrated into a final protocol for future automated production of [^18^F]RP‐115. This method holds great promise for the clinical dissemination of [^18^F]RP‐115 to evaluate patients with dementia and other diseases with dysregulated EAAT2 expression.

## 2 Methods

### 2.1 Reagents and Equipment

All reagents and solvents were purchased from Sigma‐Aldrich and used without further purification. Nuclear magnetic resonance spectroscopy was performed at the UCSF Nuclear Magnetic Resonance Laboratory on a Bruker Advance III HD 400 MHz instrument. Mass spectroscopy was performed at the University of California, Berkeley Spectrometry Facility. [^18^F]fluoride ion was generated at the UCSF Radiopharmaceutical Production Facility.

### 2.2 7‐(4,4,5,5‐Tetramethyl‐1,3,2‐Dioxaborolan‐2‐yl)‐9H‐Fluoren‐2‐Amine (1)

Miyaura borylation of 2‐amino‐7‐bromofluorene was carried out according to our previous report [28]. Briefly, 2‐amino‐7‐bromofluorene was dissolved in dioxane (15 mL), followed by the addition of [1,1′‐Bis(diphenylphosphino)ferrocene]dichloropalladium (II) (140 mg, 0.19 mmol), potassium acetate (565 mg, 5.76 mmol), and bis(pinacolato)diboron (586 mg, 2.3 mmol). The reaction mixture was stirred at 100°C for 5 h under nitrogen, with the reaction monitored by HPLC. After completion of the reaction, the mixture was cooled to room temperature, diluted with dichloromethane (30 mL), and filtered through a pad of Celite. The filtrate was washed with water (20 mL) and brine (20 mL). The collected organic layer was dried over anhydrous sodium sulfate and concentrated under reduced pressure. The crude mixture was purified by silica gel chromatography (DCM/Hexane, 1:2 to 3:1) to afford the desired product as a solid (390 mg, 66%). ^1^H NMR (400 MHz, CDCl_3_) δ 7.92 (s, 1H), 7.78 (d, *J* = 7.6 Hz, 1H), 7.63 (d, *J* = 7.6 Hz, 1H) 7.59 (d, *J* = 7.9 Hz, 1H), 6.87 (s, 1H), 6.71 (d, *J* = 8.1 Hz, 1H), 3.80 (m, 4H), 1.37 (s, 12H); ^13^C NMR (100 MHz, CDCl_3_) δ 146.4, 146.1, 145.3, 141.6, 133.6, 132.9, 131.1, 121.4, 118.1, 114.1, 111.8, 83.8, 36.7, 25.0.

### 2.3 (*S*)‐Methyl 2‐(Tert‐Butoxycarbonylamino)‐4‐(7‐(4,4,5,5‐Tetramethyl‐1,3,2‐Dioxaborolan‐2‐yl)‐9H‐Fluoren‐2‐Ylamino)‐4‐Oxo‐Butanoate (RP‐115‐Bpin, L Form)

To a stirred solution of Boc‐L‐Asp (OMe)‐OH (327 mg, 1.3 mmol) and HATU (684 mg, 1.8 mmol) in acetonitrile (9 mL) was added DIPEA (0.6 mL, 3.6 mmol). After 5 min at room temperature, compound **1** (370 mg, 1.2 mmol) was added in one portion. The reaction mixture was stirred at room temperature for 1.5 h. The solvent was removed under reduced pressure. The crude mixture was purified by silica gel chromatography (EtOAc/Hexane, 1:1) to afford the desired compound in a quantitative yield as a pale yellow solid. ^1^H NMR (400 MHz, CDCl_3_) δ 7.97 (s, 1H), 7.87 (s, 1H), 7.81 (d, *J* = 7.6 Hz, 1H), 7.72 (d, *J* = 8.1 Hz, 2H), 7.58 (s, 1H), 7.35 (dd, *J* = 8.3, 1.9 Hz, 1H), 5.75 (d, *J* = 7.2 Hz, 1H), 4.60 (dt, *J* = 8.8, 4.4 Hz, 1H), 3.87 (s, 2H), 3.79 (s, 3H), 3.12 (dd, *J* = 16.0, 4.4 Hz, 1H), 2.94 (dd, *J* = 16.0, 4.4 Hz, 1H), 1.45 (s, 9H), 1.37 (s, 12H); ^13^C NMR (100 MHz, CDCl_3_) δ 172.0, 168.3, 155.9, 145.2, 144.2, 142.5, 138.2, 136.9, 133.6, 131.3, 120.8, 119.1, 118.7, 117.0, 83.9, 80.4, 53.0, 50.6, 39.5, 36.9, 28.4, 25.0. MS (EI‐Positive) m/z [M + H]^+^ calcd for C29H38BN2O7^+^ 537.2766, found 537.2767.

### 2.4 (*R*)‐Methyl 2‐(Tert‐Butoxycarbonylamino)‐4‐(7‐(4,4,5,5‐Tetramethyl‐1,3,2‐Dioxaborolan‐2‐yl)‐9H‐Fluoren‐2‐Ylamino)‐4‐Oxo‐Butanoate (RP‐115‐Bpin, D Form)

The enantiomer of RP‐115‐Bpin was synthesized as described above, using Boc‐D‐Asp (OMe)‐OH instead of Boc‐L‐Asp (OMe)‐OH to afford the product as a white foam. ^1^H NMR (400 MHz, CDCl_3_) δ 8.68 (s, 1H), 7.97 (s, 1H), 7.86 (s, 1H), 7.82 (d, *J* = 7.6 Hz, 1H), 7.73 (d, *J* = 7.2 Hz, 2H), 7.40 (dd, *J* = 8.3, 2.0 Hz, 1H), 5.91 (s, 1H), 4.67 (s, 1H), 3.87 (s, 2H), 3.74 (s, 3H), 3.07 (dd, *J* = 17.2, 4.3 Hz, 1H), 2.77 (dd, *J* = 17.1, 6.4 Hz, 1H), 1.50 (s, 9H), 1.37 (s, 12H). ^13^C NMR (100 MHz, CDCl_3_) δ 172.8, 168.9, 155.9, 145.2, 144.3, 142.6, 138.2, 137.0, 133.6, 131.3, 120.8, 119.1, 118.8, 116.9, 83.9, 81.1, 52.4, 51.5, 37.9, 35.6, 28.5, 25.0. MS (EI‐Positive) m/z [M + H]^+^ calcd for C29H38BN2O7^+^ 537.2766, found 537.2770.

### 2.5 (*S*)‐Methyl 4‐((9H‐Fluoren‐2‐yl)amino)‐2‐((Tert‐Butoxycarbonyl)amino)‐4‐Oxobutanoate (RP‐115‐HBoc)

The coupling reaction of 2‐aminofluorene with Boc‐L‐Asp (OMe)‐OH was performed following the same procedure described for the preparation of RP‐115‐Bpin to afford the desired Boc‐protected RP‐115‐H (RP‐115‐HBoc) in 88% yield (200 mg, 0.5 mmol). ^1^H NMR (400 MHz, CDCl_3_) δ 7.89 (s, 1H), 7.71 (d, *J* = 7.5 Hz, 1H), 7.69 (d, *J* = 8.2 Hz, 1H), 7.60 (s, 1H), 7.51 (d, *J* = 7.4 Hz, 1H), 7.35 (t, *J* = 7.6 Hz, 1H), 7.32 (dd, *J* = 8.2, 2.0 Hz, 1H), 7.27 (t, *J* = 7.1 Hz, 1H), 5.77 (d, *J* = 8.3 Hz, 1H), 4.61 (dt, *J* = 8.8, 4.6 Hz, 1H), 3.86 (s, 2H), 3.80 (s, 3H), 3.13 (dd, *J* = 15.4, 4.6 Hz, 1H), 2.94 (dd, *J* = 16.0, 4.5 Hz, 1H) 1.45 (s, 9H); ^13^C NMR (100 MHz, CDCl_3_) δ 28.5, 37.1, 39.5, 50.6, 53.0, 76.8, 77.2, 77.5, 80.4, 117.1, 118.7, 119.7, 120.2, 125.1, 126.5, 126.9, 136.4, 138.4, 141.3, 143.3, 144.5, 155.9, 168.3, 172.0. MS (EI‐Positive) m/z [M + H]^+^ calcd for C23H27N2O5^+^ 411.1914, found 411.1915.

### 2.6 (*S*)‐Methyl 4‐((9H‐Fluoren‐2‐yl)amino)‐2‐Amino‐4‐Oxobutanoate (RP‐115‐H)

TFA‐mediated Boc deprotection of RP‐115‐HBoc was carried out using 50% TFA/DCM at room temperature for 2 h to afford RP‐115‐H in 86% yield (130 mg, 0.48 mmol). ^1^H NMR (400 MHz, CDCl_3_) δ 9.62 (s, 1H), 7.92 (s, 1H), 7.74 (d, *J* = 7.4 Hz, 1H), 7.71 (d, *J* = 8.2 Hz, 1H), 7.53 (d, *J* = 7.4 Hz, 1H), 7.40 (d, *J* = 8.4 Hz, 1H), 7.37 (t, *J* = 8.0 Hz, 1H), 7.29 (t, *J* = 7.0 Hz, 1H), 3.94 (dd, *J* = 9.1, 3.5 Hz, 1H), 3.90 (s, 2H), 3.80 (s, 3H), 2.86 (dd, *J* = 15.9, 3.5 Hz, 1H), 2.70 (dd, *J* = 15.8, 9.1 Hz, 1H), 1.92 (s, NH_2_); ^13^C NMR (100 MHz, CDCl_3_) δ 174.6, 168.5, 144.4, 143.3, 141.5, 138.0, 137.0, 126.9, 126.4, 125.1, 120.2, 119.6, 118.7, 117.0, 52.8, 51.5, 40.5, 37.2. MS (EI‐Positive) m/z [M + H]^+^ calcd for C18H19N2O3^+^ 311.1390, found 311.1387.

### 2.7 Copper‐Mediated Radiofluorination of [^18^F]RP115

CMRF of [^18^F]RP‐115 was carried out as described by Kaur et al. [31]. with minor modifications. Briefly, [^18^F]fluoride ion was trapped on an anion exchange cartridge (Myja Scientific) preconditioned with water (10 mL). The loaded [^18^F]fluoride ion was eluted with a solution of TEAHCO_3_ (1.5 mg) dissolved in 90% MeCN/water (1 mL). After azeotropically drying with MeCN under a vacuum/nitrogen stream at 95°C, a solution of RP‐115‐Bpin (14 μmol) and Cu (OTf)_2_Py_4_ (14 μmol) dissolved in 1,3‐dimethyl‐2‐imidazolidinone (DMI, 600 μL) was added and stirred at room temperature for 2 min, followed by heating at 115°C for 15 min. The resulting mixture was diluted with 20 mL of water and loaded onto a tC18 Sep‐Pak cartridge (Waters, preconditioned with 8 mL of EtOH and 20 mL of water), followed by washing with additional water (10 mL). The loaded mixture was eluted with 1.5 mL of TFA and stirred at room temperature for 10 min. After TFA‐mediated Boc deprotection, the mixture was diluted with water (20 mL) and passed through a preconditioned tC18 Sep‐Pak Cartridge. The cartridge was washed with additional water (10 mL), and the resulting activity was eluted with MeCN (1 mL). After dilution with water (1 mL), the crude mixture was injected into a Shimadzu LC‐40D HPLC system (Phenomenex, Luna 10 μm C18 column, 250 × 10 mm; 25% MeCN/water containing 0.1% TFA; *λ* = 280 nm, flow rate = 6.0 mL/min) to collect [^18^F]RP‐115 (*T*_*R*_ = 29 min). The radiochemical purity and identity were confirmed by Waters 590 HPLC system (Phenomenex, Luna 5 μm C18 column, 150 × 4.6 mm; 30% MeCN/water containing 0.1% TFA; *λ* = 280 nm, flow rate = 1.0 mL/min). The collected [^18^F]RP‐115 (typically 10 mL) was further diluted with 15 mL of water and loaded onto the tC18 Sep‐Pak, followed by washing with additional water (10 mL). The trapped [^18^F]RP‐115 was eluted with EtOH (1.5 mL) into a vial containing 18.5 mL of water with 0.8 mg of ascorbic acid. Molar activity and the amount of RP‐115‐H were determined using an Agilent 1200 series HPLC system (Phenomenex, Luna 5 μm C18 column, 150 × 4.6 mm; 30% MeCN/water containing 0.1% TFA; *λ* = 280 nm, flow rate = 1.0 mL/min). To determine enantiomeric purity, chiral analytical HPLC was performed by Waters 590 HPLC system (Phenomenex, Luna 5 μm Cellulose‐1 column, 50 × 4.6 mm; 20% MeCN/water containing 0.1% TFA; *λ* = 280 nm, flow rate = 1.0 mL/min). The chromatographic resolution was determined using the equation: *R*_*S*_ = 1.176 × (*t*_*R*2_ − *t*_*R*1_)/(*W*_0.5,2_ + *W*_0.5,1_), where *t*_*R*_ and *W*_0.5_ indicate the retention time and peak width at half‐height of each enantiomer (2: D‐form, 1: L‐form).

## 3 Results

With the aim of improving radiochemical yield and reducing total synthesis time of [^18^F]RP‐115, we designed a borylated RP‐115 precursor for late‐stage ^18^F‐fluorination (Figure 2A). 2‐Amino‐7‐bromo‐fluorene underwent a Pd‐catalyzed Miyaura borylation to afford compound **1** [32]. The reaction was conducted under general conditions using bis(pinacolato)diboron in the presence of Pd (dppf)Cl_2_ and potassium acetate in dioxane at 100°C. The desired borylated product was obtained in 66% isolated yield after silica‐gel column purification with high chemical purity. The formation of the borate ester was confirmed by NMR analysis (Figure S1). Subsequently, compound **1** was coupled with a protected L‐aspartic acid (Boc‐L‐Asp(OMe)‐OH) using HATU and DIPEA to afford the precursor of [^18^F]RP115 (RP‐115‐Bpin, L form) in a quantitative isolated yield. Similarly, the corresponding D‐enantiomer of RP‐115‐Bpin (D form) was synthesized using Boc‐D‐Asp(OMe)‐OH as a reference for chiral HPLC analysis of [^18^F]RP‐115. RP‐115‐H was synthesized as a reference standard to estimate the amount in the final formulated solution. 2‐Aminofluorene was coupled with Boc‐L‐Asp(OMe)‐OH, followed by TFA‐mediated Boc deprotection to afford RP‐115‐H in a quantitative yield (Figure 2B).

**FIGURE 2:**
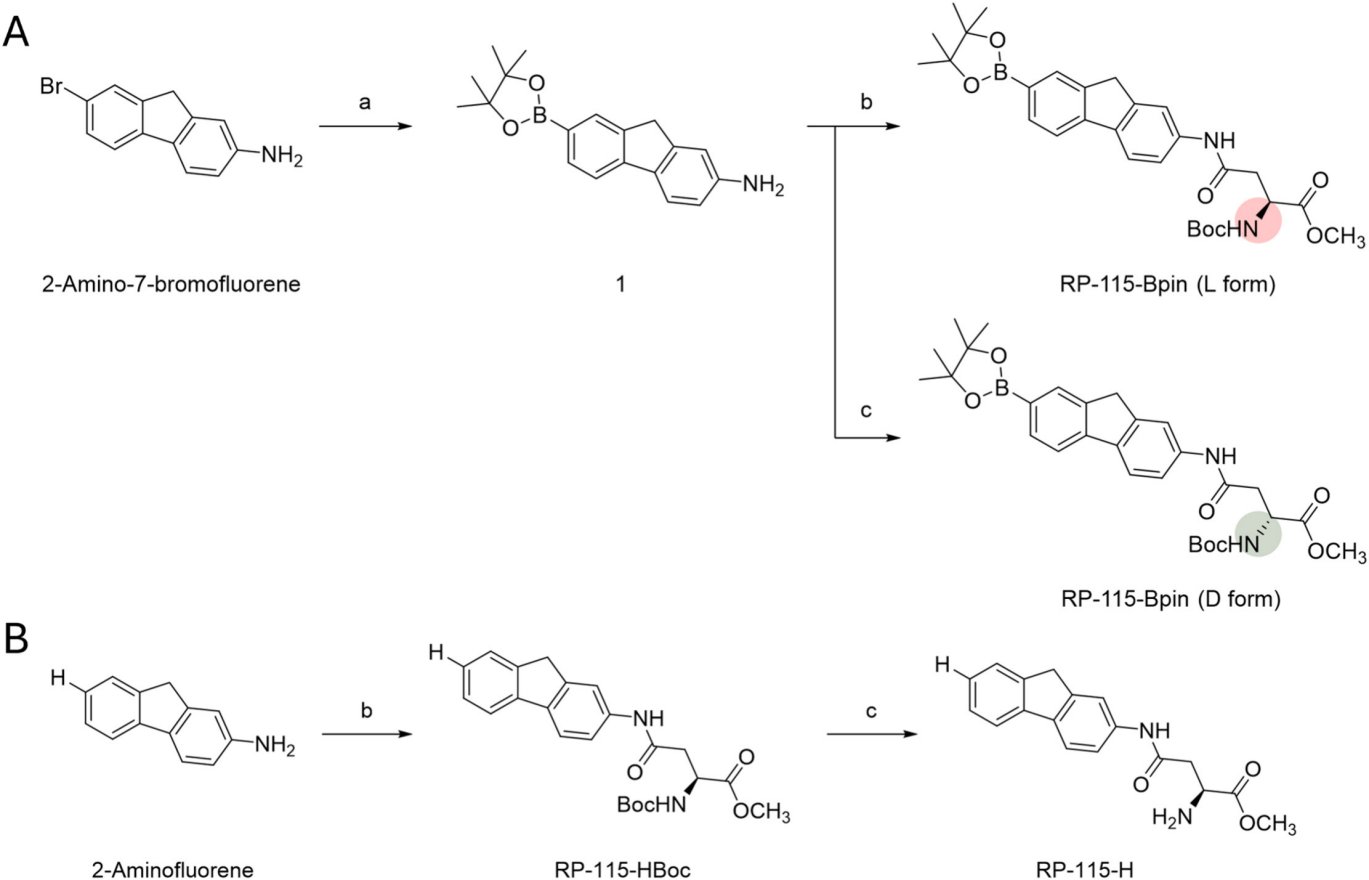
Scheme for the preparation of RP‐115‐Bpin and RP‐115‐H. (A) Synthesis of RP‐115‐Bpin starting from 2‐amino‐7‐bromofluorene. Reagents and conditions: (a) 2‐amino‐7‐bromofluorene (1 eq), Pd (dppf)_2_Cl_2_ (0.1 eq), B_2_Pin_2_ (1.2 eq), KOAc (3.0 eq), dioxane, 100°C for 5 h. (b) Compound **1** (1.0 eq), HATU (1.5 eq), DIPEA (3.0 eq), Boc‐L‐Asp (OMe)‐OH (1.1 eq), MeCN, r.t., 1.5 h. (c) Compound **1** (1.0 eq), HATU (1.5 eq), DIPEA (3.0 eq), Boc‐D‐Asp (OMe)‐OH (1.1 eq), MeCN, r.t., 1.5 h. (B) Synthesis of RP‐115‐H starting from 2‐aminofluorene. Reagent and conditions: (b) 2‐aminofluorene (1.0 eq), HATU (1.5 eq), DIPEA (3.0 eq), Boc‐L‐Asp (OMe)‐OH (1.1 eq), MeCN, r.t., 1.5 h. (c) RP‐115‐HBoc, 50% TFA/DCM, r.t., 2 h.

The radiosynthesis of [^18^F]RP‐115 was designed as a two‐step process consisting of a CMRF (step 1), followed by a TFA‐mediated Boc deprotection (step 2) (Figure 1C). We investigated each reaction step individually using HPLC purification (Figure 3A–D). [^18^F]fluoride ion (no‐carrier‐added) was loaded onto an anion‐exchange cartridge (Myja Scientific) and eluted with TEAHCO_3_ dissolved in 90% MeCN (1 mL). The loading and elution efficiency of [^18^F]fluoride ion were 97.6% ± 1.2 % and 94.3% ± 1.4 %, respectively (8.0% ± 2.5% loss of activity, *n* = 3). After azeotropic drying of [^18^F]TEAF solution, a mixture of RP‐115‐Bpin (14 μmol) and Cu (OTf)_2_Py_4_ (14 μmol) in DMI (600 μL) was added and stirred at room temperature for 2 min, followed by heating at 115°C for 15 min. The reaction mixture was loaded onto a tC18 cartridge and eluted with MeCN. The loading and elution efficiency were 75.1% ± 7.3 % and 94.3% ± 2.0 %, respectively. The eluted activity was injected onto the HPLC column. As shown in the representative HPLC profile, RP‐115‐H‐Boc (protonated form, *T*_*R*_ = 24.8 min) eluted prior to [^18^F]RP‐115‐Boc (*T*_*R*_ = 27.1 min) (Figure 3A,B).

**FIGURE 3:**
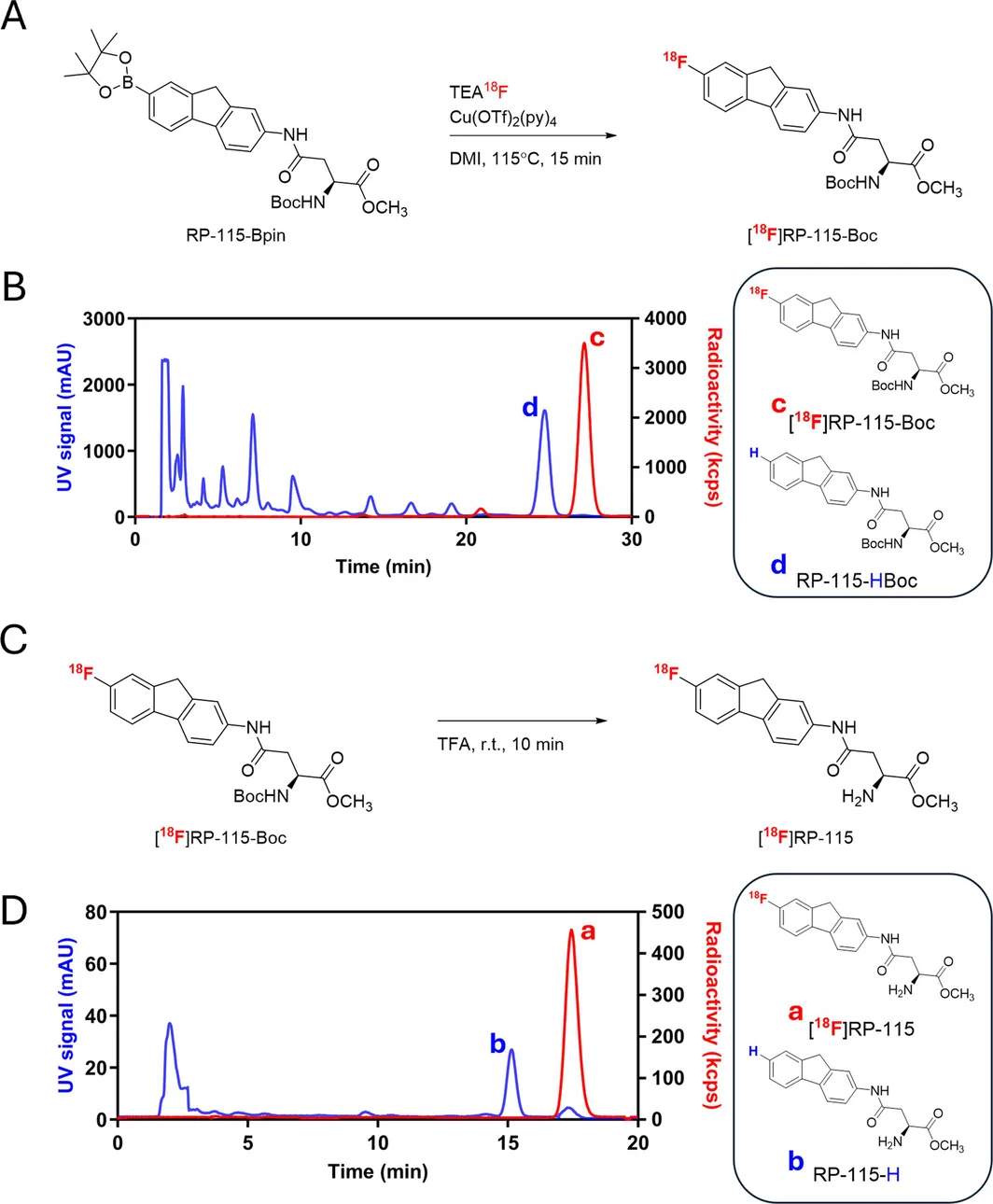
Systematic investigation of the two‐step radiosynthesis of [^18^F]RP‐115. (A) Copper‐mediated radiofluorination of RP‐115‐Bpin. (B) Representative HPLC profile of the crude mixture after [^18^F]fluorination. (C) TFA‐mediated Boc deprotection of [^18^F]RP‐115‐Boc. (D) Representative HPLC profile of the crude mixture after deprotection.

The collected [^18^F]RP‐115‐Boc was concentrated using a tC18 Sep‐Pak cartridge, with less than 1% loss of activity observed during trapping. The trapped activity was eluted with TFA with 94.8% ± 1.7 % of elution efficiency. After TFA‐mediated Boc deprotection, the mixture was loaded on tC18 Sep‐Pak, with quantitative (> 99%) efficiency and the elution efficiency was 96.2% ± 1.1%. After tC18 cartridge purification, the resulting mixture was purified by reverse‐phase HPLC. As shown in the representative HPLC profile, [^18^F]RP‐115 eluted at 17.5 min. The UV signal observed at 15.1 min was assigned to RP‐115‐H. Although most of the protonated form was effectively removed during the first HPLC purification step, the remaining amount was still greater than the mass of [^18^F]RP‐115, indicating a potential impact on the effective molar activity (Figure 3C,D).

Finally, radiosynthesis of [^18^F]RP‐115 (Figure 4A) was carried out through the following sequence: (1) CMRF, (2) solvent exchange using a tC18 Sep‐Pak cartridge, (3) TFA‐mediated Boc deprotection, (4) solvent exchange through tC18 Sep‐Pak cartridge, (5) HPLC purification, and (6) final formulation. To minimize potential contamination of RP‐115‐H in the final formulation (Figure 3D; 30% v/v, containing 0.1% TFA), a slightly lower concentration of MeCN/Water (25% v/v, containing 0.1% TFA; Figure 4B) was adopted as the mobile phase. As shown in the representative HPLC profile, RP‐115‐H eluted at 20 to 25 min, whereas [^18^F]RP‐115 eluted later, between 27 and 30 min, indicating improved chromatographic resolution (Figure 4B). The identity of [^18^F]RP‐115 was confirmed by co‐injection with nonradioactive ^19^F standard RP‐115 (Figure 4C). The collected [^18^F]RP‐115 was diluted with water (15 mL), loaded onto a tC18 Sep‐Pak, and further washed with additional water (10 mL). The trapped [^18^F]RP‐115 was subsequently eluted with 1.5 mL of EtOH and formulated with 18.5 mL of water containing 0.8 mg of ascorbic acid. The decay corrected radiochemical yield of [^18^F]RP‐115 was 52.8 ± 2.1 % (based on the initial [^18^F]fluoride ion, *n* = 4; 34.6% ± 4.0% non‐decay corrected) with high radiochemical purity (> 99%) within a total synthesis time of approximately 70 min. Calibration curves were generated using nonradioactive ^19^F standard RP‐115 and RP‐115‐H to calculate molar activity and estimate the amount of protonated form in the final formulation (Figures S2 and S3). The calculated molar activity of [^18^F]RP‐115 was 355 ± 61 GBq/μmol at the end of synthesis. To determine the molar activity, an aliquot (30 μL) of the final formulated solution was injected to an analytical HPLC. The molar activity was calculated by dividing the radioactivity of the injected volume by the amount of nonradioactive RP‐115, derived from a calibration curve. Although trace amounts of RP‐115‐H were detected by analytical HPLC (the limit of detection = 0.05 nmol/mL), its concentration was below the limit of quantification (< 0.15 nmol/mL) in the final formulation (Figure 5A,B).

**FIGURE 4:**
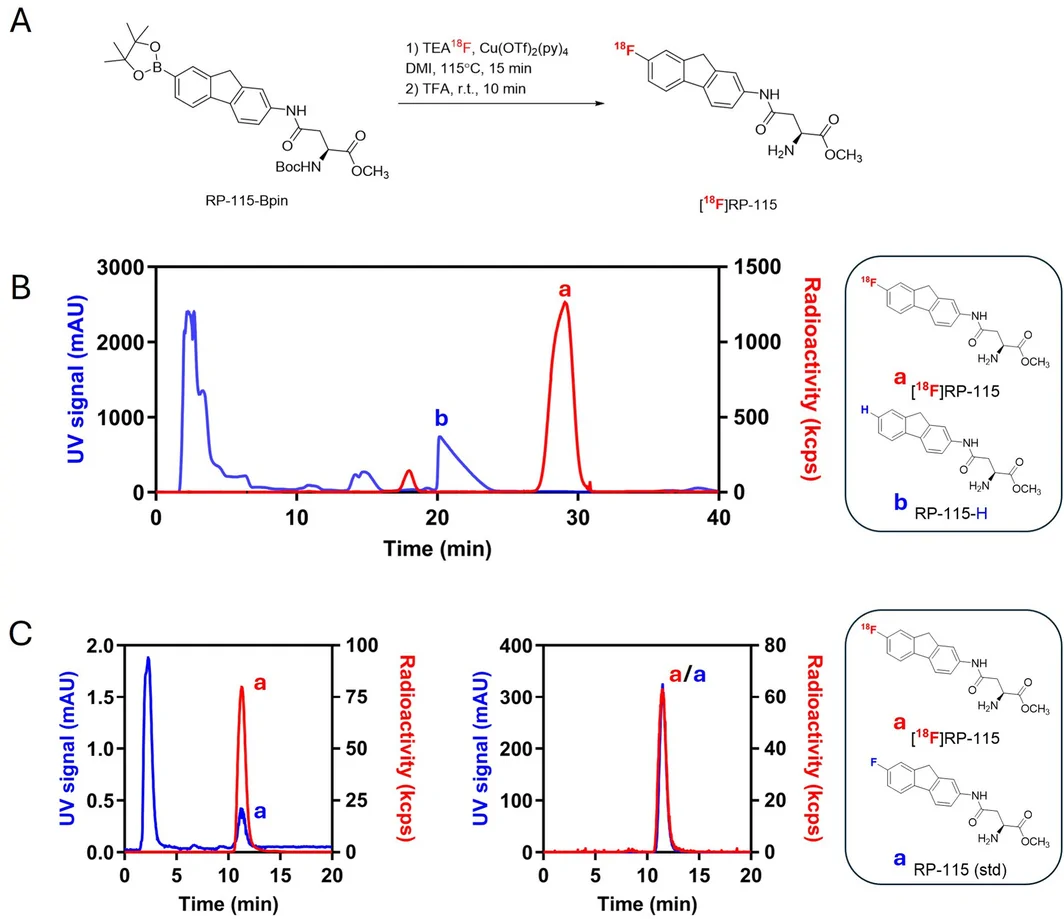
Radiosynthesis of [^18^F]RP‐115 with a single HPLC purification. (A) Scheme for the radiosynthesis of [^18^F]RP‐115 starting from RP‐115‐Bpin. (B) Representative HPLC profile of the crude mixture after [^18^F]fluorination, followed by deprotection. (C) Representative analytical HPLC profile of [^18^F]RP‐115 after HPLC purification (left). The identity of [^18^F]RP‐115 was confirmed by coinjection with nonradioactive RP‐115 standard (right).

**FIGURE 5:**
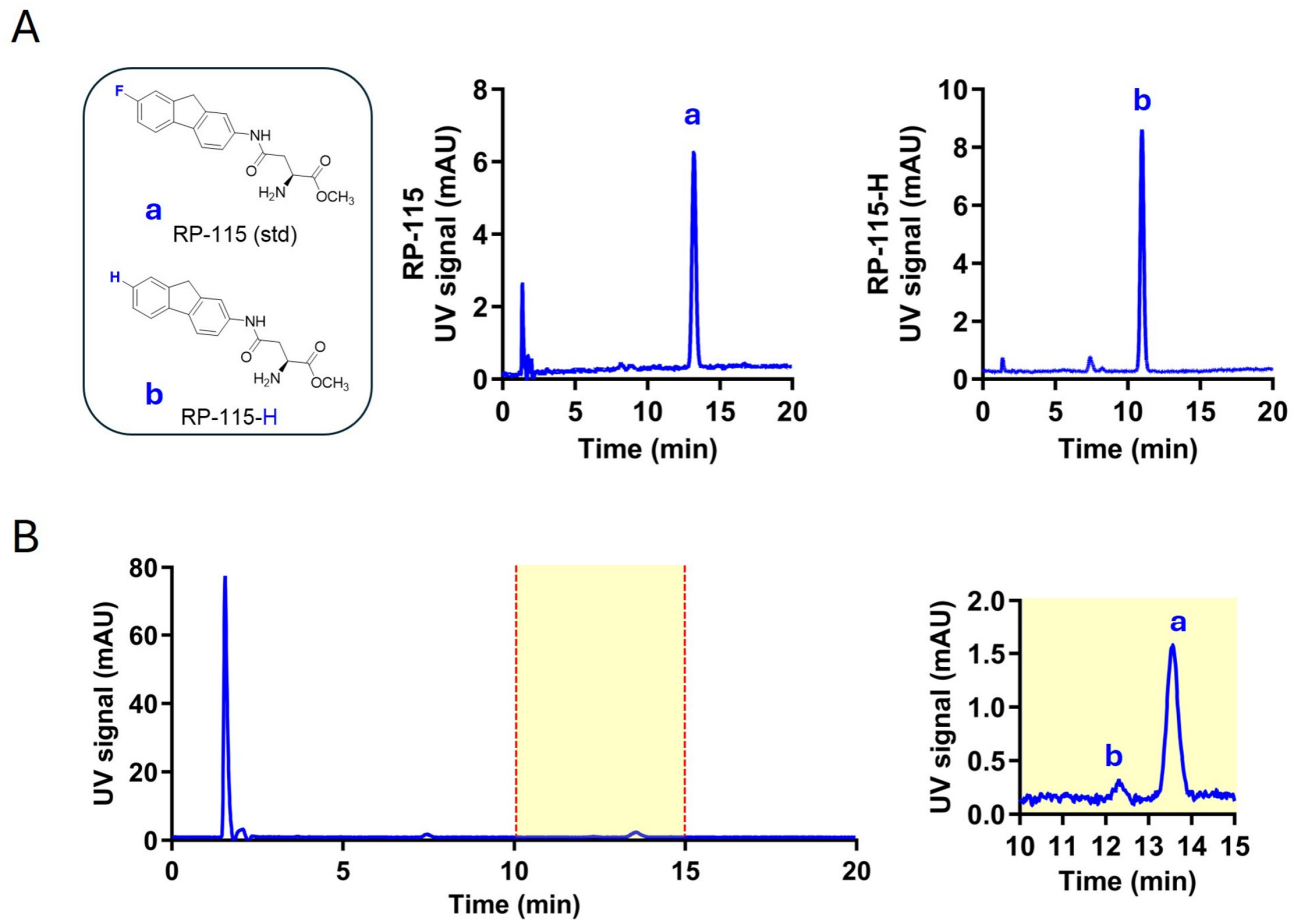
Analytical HPLC analysis for assessment of impurities (RP‐115‐H) in the final [^18^F]RP‐115 formulation. (A) Structures and analytical HPLC profiles of RP‐115 (a) and RP‐115‐H (b) standards. (B) Analytical HPLC profile of the final RP‐115 formulation. The yellow highlighted region is enlarged to highlight the detection of RP‐115 (a) and RP‐115‐H (b).

To investigate whether racemization occurred during the CMRF and TFA‐mediated deprotection, chiral HPLC analysis was performed using a Lux Cellulose‐1 column (Figure 6A–C). The D‐enantiomer of [^18^F]RP‐115 was prepared from the corresponding RP‐115‐Bpin (D form) as a reference (Figure 6A). The desired [^18^F]RP‐115 (L form) eluted at a retention time of 5.9 min (Figure 6B, left panel). The chemical identity and enantiomeric purity were confirmed by co‐injection with the RP‐115 std. (L form), as shown in Figure 6B (right panel). Under the same analysis conditions, the control D form showed a retention time of 7.8 min (Figure 6C, left panel). Finally, co‐injection of both the L‐enantiomer (target compound, [^18^F]RP‐115) and D‐enantiomer (control) showed an enantiomeric separation resolution on the Lux Cellulose‐1 column (Figure 6C, right panel). The chromatographic resolution (*R*_*S*_) between the two enantiomers was determined to be 1.51, indicating baseline separation (*R*_*S*_ > 1.5) under the analytical HPLC conditions. Collectively, these data indicate that the configuration of the aspartic acid residue remained intact during CMRF followed by TFA‐mediated deprotection under the optimized conditions of [^18^F]RP‐115 radiosynthesis.

**FIGURE 6:**
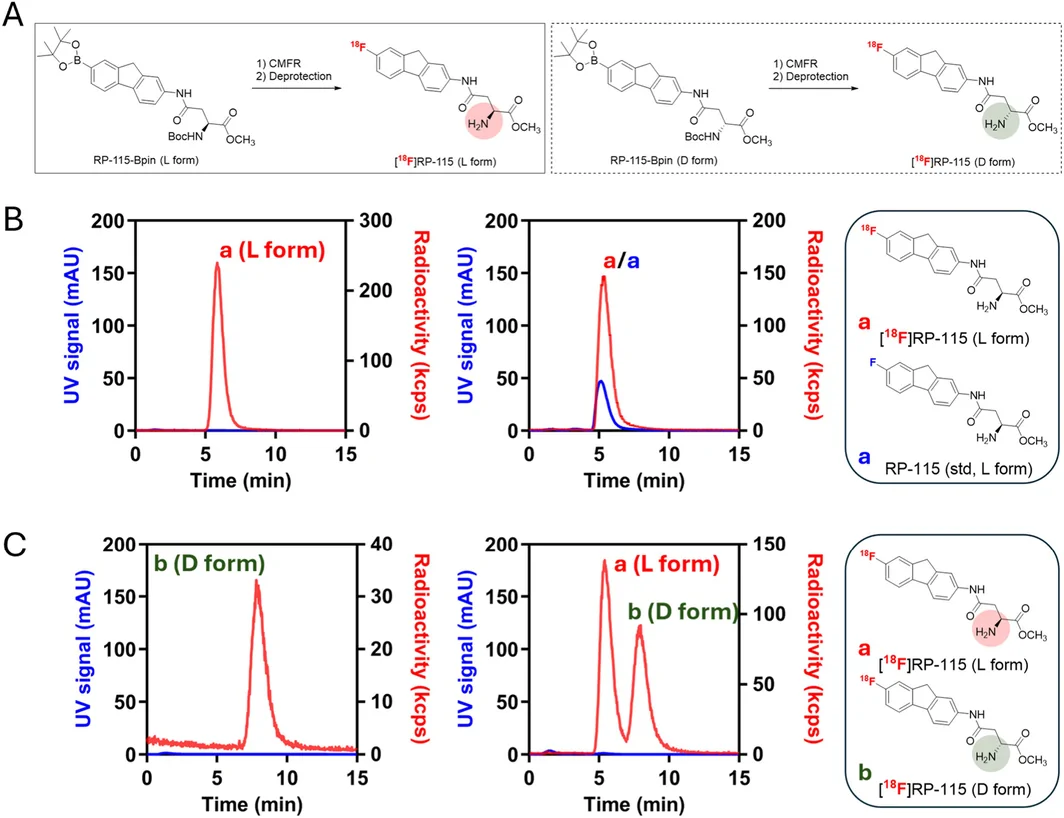
Radiosynthesis and chiral HPLC analysis of [^18^F]RP‐115. (A) Scheme for the radiosynthesis of [^18^F]RP‐115 (L‐form, left scheme; D‐form, right scheme) starting from their corresponding RP‐115‐Bpin precursor (L or D form). (B) Chiral HPLC profile of [^18^F]RP‐115 (L form) after HPLC purification (left). The identity of [^18^F]RP‐115 (L form) was confirmed by coinjection with nonradioactive RP‐115 (L form) standard (right). (C) Chiral HPLC profile of [^18^F]RP‐115 (D form) after HPLC purification (left). The right panel shows the coinjection of two L‐ and D‐enantiomers of [^18^F]RP‐115, indicating enantiomeric separation using the Lux Cellulose‐1 column.

## 4 Discussion

In this work, we developed an alternative radiosynthesis of [^18^F]RP‐115 via CMRF using the RP‐115‐Bpin precursor starting from 2‐amino‐7‐bromofluorene via borylation and coupling with a protected L‐aspartic acid. Our initial attempt was the coupling of 2‐amino‐7‐bromofluorene with the protected L‐aspartic acid, Boc‐L‐Asp (OMe)‐OH, followed by Pd‐catalyzed Miyaura borylation. Although the coupling step proceeded quantitatively, the subsequent borylation of RP‐115‐Br afforded a mixture containing unreacted starting material and an impurity (RP‐115‐H), which was challenging to separate by manual silica‐gel column chromatography (Figure S4) [30]. To circumvent this purification issue, the order of the synthetic steps was reversed by performing the borylation first, followed by coupling with the aspartic acid residue. To facilitate purification after borylation, the reaction was monitored by HPLC until the bromo compound was completely consumed. Although the starting material, 2‐amino‐7‐bromofluorene (90% purity; Aldrich), was found to include a small amount of 2‐aminofluorene as an impurity, subsequent purification via silica gel column chromatography successfully afforded the borylated fluorene in high chemical purity (Figure S5). Finally, coupling this high purity of the borylated fluorene with the protected L‐aspartic acid allowed us to obtain the RP‐115‐Bpin precursor in high chemical purity (Figure S6). Although CMRF has emerged as a promising technique for ^18^F‐labeling, challenges regarding the synthesis and stability of boronated precursors typically require strategic synthesis and expertise. To address these challenges, a method for generating ^18^F‐tracers directly from (hetero)aryl halides via sequential Miyaura borylation/CMRF was recently reported [30]. This in situ two‐step synthesis offers a significant practical advantage on CMFR workflow by bypassing the need to isolate, purify, or store sensitive organoboron precursors, which may improve the accessibility and synthetic efficiency of [^18^F]RP‐115.

Meanwhile, protodemetalation is a competing side reaction commonly observed in CMRF [33]. For small and relatively lipophilic radiotracers targeting the brain, HPLC purification is typically performed using reversed‐phase C18 chromatography. The desired fluorine‐18 labeled product is more lipophilic than its corresponding protonated byproduct, and therefore elutes closely, but slightly later under reversed‐phase C18 column conditions. Consequently, the desired product may contain a small amount of the earlier eluting protonated analog if purification is not carefully optimized [34]. The amount of the protonated analog is of particular concern, as it may possess binding affinity like that of the fluorine‐18 labeled molecule. Such contamination could ultimately affect imaging outcomes, particularly in brain PET scans, where BBB penetration and EAAT2 sites may be limited. In our study, although trace amounts of RP‐115‐H were detected by analytical HPLC, its concentration was below 0.15 nmol/mL (below the limit of quantification). However, because radioactive decay exponentially decreases effective molar activity over time, such trace protonated impurities remain a concern. Future optimization may consider reported methods to mitigate protodeborylation or alternative purification column (e.g., perfluorophenyl‐capped HPLC column) to resolve this concern [35].

It should be noted that while previous multistep method was fully optimized using automated synthesis module, the CMRF‐based simplified method reported in this study was performed manually. Thereby, a direct comparison of overall practical efficiency remains limited at this stage. Nevertheless, the efficient radiosynthesis demonstrated in our manual two‐step synthesis supports its feasibility as an alternative synthetic route for the future automated production of EAAT2‐targeting PET tracer [^18^F]RP‐115. Future studies will focus on optimizing this simplified method on an automation module to enable a direct comparison of its practical efficiency with existing methods for the production of [^18^F]RP‐115.

## 5 Conclusion

We established a CMRF‐based radiosynthesis of [^18^F]RP‐115 using RP‐115‐Bpin precursor. The CMRF reaction proceeded efficiently on the relatively electron‐rich fluorene scaffold, which may be challenging for conventional aromatic fluorination, resulting in high radiochemical yield of [^18^F]RP‐115 without any detectable racemization. This method has several potential advantages, including enhanced radiochemical yield, molar activity, and total synthesis time, facilitating clinical studies. Although substantial protodemetallation was observed after CMRF, representative HPLC profiles of the crude mixture and final formulation indicated that the desired [^18^F]RP‐115 product could be readily separated from the protonated byproduct RP‐115‐H using isocratic reversed‐phase HPLC. Based on this step‐by‐step investigation, future work will focus on implementing this CMRF‐based automated radiosynthesis of [^18^F]RP‐115 and comparing this method to the multistep approach previously reported.

## Author Contributions

D.M.W. supervised the overall project. S.H.L., H.F.V., and D.M.W. conceptualized and designed the study. S.H.L., J. B, and J.M.K. performed chemistry and radiochemistry. S.H.L. and D.M.W. wrote the original draft of the manuscript. All authors reviewed and edited the manuscript. All authors have agreed to the publication of the manuscript and take responsibility for the work's contents.

## Funding

This study was supported by the National Institute of Health (1R01AG072743‐01A1) and Alzheimer's Drug Discovery Foundation (RC‐201810‐2017455).

## Ethics Statement

The authors have nothing to report.

## Consent

The authors have nothing to report.

## Conflicts of Interest

C.‐K.C., J.R.F., and J.M.G. are affiliated with Rio Pharmaceuticals Inc.; J.M.G. is a named inventor on US Patent 9499472. The remaining authors declare no competing interests.

## Supporting information




**Figure S1:** NMR analysis in CDCl_3_.
**Figure S2:** Calibration curve of RP‐115 to estimate molar activity of [18F]RP‐115.
**Figure S3:** Calibration curve of RP‐115‐H to estimate the amount of RP‐115‐H in the final formulation.
**Figure S4:** HPLC profile indicating the limitation of manual silica‐gel purification after Miyaura borylation.
**Figure S5:** HPLC profiles of commercial 2‐amino‐7‐bromofluorene, 2‐aminofluorene, the crude reaction mixture 5 h after the Miyaura borylation, and the purified 2‐aminofluorene‐Bpin via silica gel column chromatography.
**Figure S6:** HPLC profile demonstrating the high chemical purity of the RP‐115‐Bpin precursor from the corresponding high purity of 2‐aminofluorene‐Bpin.

## Data Availability

Additional supporting information can be found online in the Supporting Information section.
