# Supplementary material for: Copper‐Mediated Radiofluorination of the Excitatory Amino Acid Transporter 2 (EAAT2)–Targeted PET Tracer [18F]RP‐115: Alternate Synthetic Method
Source: J Labelled Comp Radiopharm. 2026 Oct 28;69(13-14):e70044. doi: 10.1002/jlcr.70044 (PMC13716918; doi:10.1002/jlcr.70044)
Supplement: Supplementary file 1 [file JLCR-69-0-s001.docx]

**Supporting Information for**

**Title:** Copper-mediated radiofluorination of the excitatory amino acid transporter 2 (EAAT2)-targeted PET tracer [^18^F]RP-115: alternate synthetic method

**Contents:**

**Supplemental Figure and Table**	page 2 - 8

Figure S1: NMR analysis in CDCl_3_.

Figure S2: Calibration curve of RP-115 to estimate molar activity of [^18^F]RP-115.

Figure S3: Calibration curve of RP-115-H to estimate the amount of RP-115-H in the final formulation.

Figure S4: HPLC profile indicating the limitation of manual silica-gel purification after Miyaura borylation.

Figure S5: HPLC profiles of commercial 2-amino-7-bromofluorene, 2-aminofluorene, the crude reaction mixture 5 hours after the Miyaura borylation, and the purified 2-aminofluorene-Bpin via silica gel column chromatography.

Figure S6: HPLC profile demonstrating the high chemical purity of the RP-115-Bpin precursor from the corresponding high purity of 2-aminofluorene-Bpin.

**Figure S1.** NMR analysis in CDCl_3_.

**7-(4,4,5,5-tetramethyl-1,3,2-dioxaborolan-2-yl)-9H-fluoren-2-amine** ^1^H NMR

**7-(4,4,5,5-tetramethyl-1,3,2-dioxaborolan-2-yl)-9H-fluoren-2-amine** ^13^C NMR

**RP-115-Bpin (L form)** ^1^H NMR

**RP-115-Bpin (L form)** ^13^C NMR

**RP-115-HBoc** ^1^H NMR

**RP-115-HBoc** ^13^C NMR

**RP-115-H** ^1^H NMR

**RP-115-H** ^13^C NMR

**RP-115-Bpin (D form)** ^1^H NMR

**RP-115-Bpin (D form)** ^13^C NMR

**Figure S2**. Calibration curve of RP-115 to estimate molar activity of [^18^F]RP-115 (R^2^ = 0.9992).

**Figure S3**. Calibration curve of RP-115-H to estimate the amount of RP-115-H in the final formulation (R^2^ = 0.9998).

**Figure S4**. HPLC (Phenomenex; Luna 10 μm C18 250 × 4.6 mm; 1 mL/min) chromatogram indicating the limitation of manual silica-gel purification after Miyaura borylation. The HPLC gradient elution was performed using water and acetonitrile (both containing 0.1% TFA): 20% acetonitrile held for 2 min, increased to 90% for 10 min, held at 90% for 2 min, and returned to 20% for 2 min followed by a 4 min re-equilibration.

**Figure S5**. HPLC (Phenomenex; Luna 10 μm C18 250 × 4.6 mm; 1 mL/min) profile of (A) commercially obtained 2-amino-7-bromofluorene (Sigma-Aldrich), (B) 2-aminofluorene, (C) the crude reaction mixture 5 hours after the Miyaura borylation, (D) the purified 2-aminofluorene-Bpin via silica gel column chromatography. The HPLC gradient elution was performed using water and acetonitrile (both containing 0.1% TFA): 20% acetonitrile held for 2 min, increased to 90% for 10 min, held at 90% for 2 min, and returned to 20% for 2 min followed by a 4 min re-equilibration.

**Figure S6**. HPLC profile demonstrating the high chemical purity of the RP-115-Bpin precursor from the corresponding high purity of 2-aminofluorene-Bpin. The HPLC gradient elution was performed using water and acetonitrile (both containing 0.1% TFA): 20% acetonitrile held for 2 min, increased to 90% for 10 min, held at 90% for 2 min, and returned to 20% for 2 min followed by a 4 min re-equilibration.
